# Disposal Situation of Sewage Sludge from Municipal Wastewater Treatment Plants (WWTPs) and Assessment of the Ecological Risk of Heavy Metals for Its Land Use in Shanxi, China

**DOI:** 10.3390/ijerph14070823

**Published:** 2017-07-21

**Authors:** Baoling Duan, Wuping Zhang, Haixia Zheng, Chunyan Wu, Qiang Zhang, Yushan Bu

**Affiliations:** 1 College of Resources and Environment, Shanxi Agricultural University, Taigu 030801, Shanxi, China; sxnddbl@163.com (B.D.); zwping@126.com (W.Z.); zhenghaixia81@126.com (H.Z.); chunyan_wu224@163.com (C.W.); 18334759065@163.com (Q.Z.); 2 College of Forestry, Shanxi Agricultural University, Taigu 030801, Shanxi, China

**Keywords:** heavy metals, sewage sludge, ecological risk, geoaccumulation index, single-factor pollution index, Nemerow’s synthetic pollution index, monomial potential ecological risk coefficient, potential ecological risk index

## Abstract

Land use of sewage sludge is the primary disposal method in Shanxi, accounting for 42.66% of all. To determine the ecological risk of heavy metals in sewage sludge, contents of seven heavy metals in sewage sludge from 9 municipal waste water treatment plants (WWTPs) that had the highest application for land use were determined. The order of the measured concentrations was: Zn > Cr > Cu > Ni > Pb > As > Cd, and all heavy metals contents were within the threshold limit values of the Chinese Control Standards for Pollutants in Sludge from Agriculture Use (GB4284-84). Four indices were used to assess the pollution and the ecological risk of heavy metals. By the mean values of the geoaccumulation index (I_geo_), heavy metals were ranked in the following order: Cd > Zn > Cu > As > Cr > Ni > Pb. The values showed that the pollution of Zn in station 3 and Cd in station 1, 2, 3, 4, 8 and 9 were heavily; Cu in station 8 and 9, Zn in station 1, 2, 4, 8 and 9 and Cd in station 5 and 7 were moderately to heavily, and the accumulation of other heavy metals were not significant. The single-factor pollution index (PI) suggested that none of the stations had heavy metals contamination, except for Cu in station 9, Zn in station 3 and 8, and Cd in station 1 and 9, which were at a moderate level. According to the results of the Nemerow’s synthetic pollution index (PN), sewage sludge from all stations was safe for land use with respect to heavy metals contamination, except for stations 3, 8 and 9, which were at the warning line. The monomial potential ecological risk coefficient ($E_{r}^{i}$) revealed that heavy metals ecological risks in most stations were low. However, station 9 had a moderate risk for Cu; station 6 had a moderate risk, stations 5 and 7 had high risk, other stations had very high risk for Cd. According to the results of the potential ecological risk index (RI), station 1, 8 and 9 had high risk; station 2, 3, 4, 5 and 7 had a moderate risk, and station 6 had a low risk. The preliminary results indicated that the potential risk of land exposure to heavy metals in sewage sludge was relatively low, with Zn and Cd as the main contributor to the ecological risk for the applying of sewage sludge on land. Additionally, stations 3, 8 and 9 require more attention regarding the land applications related to heavy metals pollution.

## 1. Introduction

The increasing sewage sludge produced by wastewater treatment plants (WWTPs) requires disposal [1,2]. After disposal at sea, land use of sewage sludge as soil conditioners or fertilizers has become the most common disposal way, owing to the abundance of valuable components such as organic matter, nitrogen, phosphorus, potassium and other nutrients for plant growth [3,4,5,6,7,8].

In America, approximately 60% of the sewage sludge is used to condition soil or as fertilizers [6]. In European Union countries, more than 50% of the sludge is disposed by land application such as France, Belgium and the United Kingdom [1,3]. The application of sewage sludge to land can increase the plant yields and enhance soil properties such as pH, organic matter, nutrients, porosity, aggregate stability, bulk density and water retention and movement [1,3,9,10]. However, sludge can also lead to environmental pollution because it contains various pollutants, especially heavy metals [11]. Heavy metals, as the limiting factor for the land use of sewage sludge, can cause harm to the soil-plant system, and furthermore, might pose a serious risk to human health [1,9,10]. To avoid this risk, China, America, Europe, and some other countries have established criteria for the concentration of heavy metals in sewage sludge used for land applications [4].

The risk assessment of heavy metals addresses the evaluation of the hazardous properties of these environmental pollutants and the exposure of the land to them. To evaluate the environmental risk of heavy metals in sewage sludge, the geo-accumulation index (I_geo_), single-factor pollution index (PI), Nemerow’s synthetic pollution index (PN) and ecological risk index (RI) were applied [12,13,14,15,16,17,18,19]. The different indices focus on different aspects: I_geo_ and PI both focus on the accumulation levels of each heavy metals in the samples, without any attention to their toxicity [1,16,17,18,19,20,21,22,23]; PN and RI, which are used to assess the comprehensive ecological risks, consider more factors than just the total quantity of the heavy metals [1,14,24]. PN focuses on the average and maximum pollution of each heavy metal [14,16,18], and RI considers the toxicity of heavy metals [1,14,17,18,19,22,23,24,25]. As such, together, they provide an overall index for the risk assessment [18,19,20,21,22,23,24,25]. Assessing the ecological risk of heavy metals in sewage sludge is vital for reducing the risk of applying sewage sludge on land [1,3,5,23].

Shanxi, located in the Loess Plateau, has poor soil [26]. It is reported that the organic matter, total nitrogen, total phosphorus, and total potassium of cultivated land in Shanxi is 10.7, 0.68, 0.65 and 18.5 g/kg, respectively, which are lower than those in other regions in China [26,27]. Sewage sludge contains abundant materials that are essential for cultivating plants. Using sewage sludge on land not only relieves the impoverishment of the soil, but also offers an effective means to dispose of massive quantities of sludge. Because the output of sewage sludge had reached 428.4 t/d in Shanxi by 2012, the potential for sewage sludge use on land is high [26]. To use sewage sludge in an environmentally safe manner, a risk assessment should be implemented, and the Chinese Control Standards for Pollutants in Sludge from Agricultural Use (GB4284-84) which is the present executive standard in China to control heavy metals contents when sewage sludge used on land, is the foundation of this study. The goals of this study are to: (a) Survey the situation of sewage sludge disposal in Shanxi; (b) measure heavy metals concentration in the sewage sludge collected from different WWTPs in Shanxi; and (c) assess the ecological risk of heavy metals in sewage sludge for land use.

## 2. Disposal Situation of Sewage Sludge in Shanxi

In Shanxi, 157 WWTPs were constructed in 2011, which produced 374,859.15 tons of sewage sludge, as showed in Table 1. Land use, land fill, construction matter production, burning and dumping are the five methods for disposing of sewage sludge, with the quantity of each method being 159,910.2, 182,976.25, 12,749.7, 19,201.5, and 21.5 tons, respectively. Land use and land fill are the primary methods, representing 42.66% and 48.81%, with the other three methods accounting for 8.52%. Different regions have employed different disposal methods for sewage sludge, and the situation is illustrated in Table 1: land fill was the primary method in the north, as much as 85.9%; land use was the main method in the middle region, accounting for 72.9% of the total; in the south, land fill was the principal method, with land use and construction matter production complementing; land fill was the main method, and land use was as one other method of sewage sludge disposal in the south east of Shanxi. Although the different regions use different methods to dispose of sewage sludge, land use and land fill were the main methods of handling the increasing amount of sewage sludge.

## 3. Materials and Methods

### 3.1. Sampling

**Figure 1:**
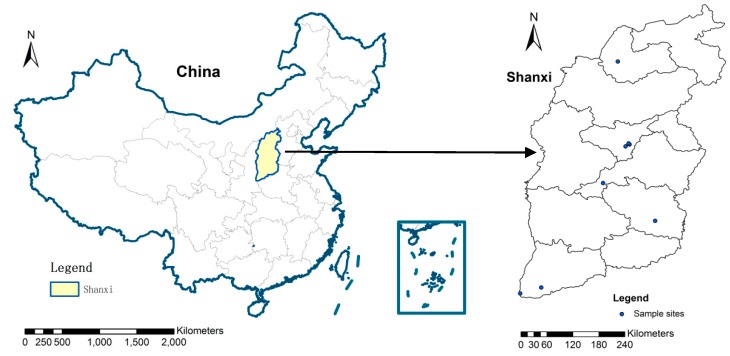
Sampling sites of wastewater treatment plants (WWPTs) locations in Shanxi Province, China.

To assess the risk of heavy metals in sewage sludge that is disposed through land use, 9 municipal WWTPs, with a daily output greater than 5000 tons in Shanxi Province, China, were selected to collect sewage sludge, as indicated in Figure 1. To enhance the representativeness of the samples, at each WWPT four subsamples were collected from different sites in the storage pile, and the four subsamples from the same WWTP were mixed together as one sample.

### 3.2. Determination of the Total Heavy Metal Concentration

The collected samples were dried at room temperature, grounded, and homogenized in an agate mortar; then, they were sieved through a sieve (mesh pore size: 0.14 mm), and stored in jars at room temperature. Dry sludge samples were weighted and digested with HNO_3_, using a microwave digestion system (Mars 5, CEM, Saint Matthews, NC, USA) as per procedure of the United States Environmental Protection Agency Method 3051B [28]. Pb, Cu, Zn, Ni and Cr were analyzed using an atomic absorption spectrophotometer (VARIAN-AA-240, VARIAN, Palo Alto, CA, USA); As was analyzed using an atomic fluorescence spectrometer (AFS-230E, Haiguang, Beijing, China) and Cd was analyzed using a graphite furnace atomic absorption spectrophotometer (TAS-990AFG, PERSEE, Beijing, China). The standard reference sludge samples (RTC-CRM055, TMRM, Shanghai, China) and national standards of China (GB/T 15555.2-1995, GB/T 15555.2-1995, GB/T 15555.2-1995, GB/T 15555.9-1995, GB/T 15555.6-1995, GB/T 22105.2-2008 and GB/T 17141-1997) were used for quality control. Triplicate samples were determined, and the mean values of the results were reported as the final concentrations of the heavy metals.

### 3.3. Geoaccumulation Index (I_geo_)

The geoaccumulation index (I_geo_) had been defined in the 1960s to assess the pollution of heavy metals in bottom sediments [29]. This index evaluates the contamination of trace metals by comparing the current contents with pre-industrial values. I_geo_ was defined as follows [29]:

$$I_{\mathrm{geo}}=\log_{2}\left(C_{n}/1.5B_{n}\right)$$

where C_n_ is the content of heavy metal (n) in samples, mg/kg; B_n_ is the background concentration of the metal (n), using the Chinese geochemical background concentration of heavy metal (n); 1.5 is a constant factor due to the lithospheric effects. To determination the level of contamination, Muller defined seven classes of pollution by I_geo_: Class 0 ($I_{\mathrm{geo}}$ ≤ 0): practically unpolluted; Class 1 (0 < $I_{\mathrm{geo}}$ ≤ 1): unpolluted to moderately polluted; Class 2 (1 < $I_{\mathrm{geo}}$ ≤ 2): moderately polluted; Class 3 (2 < $I_{\mathrm{geo}}$ ≤ 3): moderately to heavily polluted; Class 4 (3 < $I_{\mathrm{geo}}$ ≤ 4): heavily polluted; Class 5 (4 < $I_{\mathrm{geo}}$ ≤ 5): heavily to extremely polluted; Class 6 ($I_{\mathrm{geo}}$ > 5): extremely polluted [23].

### 3.4. Assessment of Heavy Metal Pollution

The single-factor pollution index (PI) and the Nemerow’s synthetic pollution index (PN) were defined to evaluate the presence of a single heavy metal, and to assess the pollution levels in the studying station.

$$\mathrm{PI}=C_{i}/S_{i}$$

where PI is the single-factor pollution index of the ith heavy metal; $C_{i}$ is the concentration of the ith heavy metal, mg/kg; $S_{i}$ is the standard of the ith heavy metal, mg/kg, according to Chinese Control Standards for Pollutants in Sludge from Agricultural Use (GB4284-84, Ph ≥ 6.5), the corresponding standard values ($S_{i}$) for Pb, Cu, Zn, Ni, Cr, As and Cd are 300, 250, 500, 100, 600, 75 and 5 mg/kg respectively [30]. The pollution status of heavy metals is classified into five levels based on the corresponding PI values: no contamination (PI ≤ 1.0), low level of contamination (1.0 < PI ≤ 2.0), moderate level of contamination (2.0 < PI ≤ 3.0), strong level of contamination (3.0 < PI ≤ 5.0) and very strong level of contamination (PI > 5.0) [16,19,31,32].

The Nemerow’s synthetic pollution index has been applied to assess heavy metal contamination, and has been widely used to reflect the total pollution level of environmental quality. This index was defined as follows [33]:

$$\mathrm{PN}=\sqrt{\frac{P_{i,\;\mathrm{ave}}^{2}+P_{i,\max}^{2}}{2}}$$

where PN is the synthetic pollution index; $P_{i,\mathrm{ave}}$ is the average value of the single-factor pollution index of the ith heavy metal; $P_{i,\max}$ is the maximum value of the single-factor pollution index of the ith heavy metal. The index is divided into 5 classes: safety (PN ≤ 0.7), warning line of pollution (0.7 < PN ≤ 1.0), slight pollution (1.0 < PN ≤ 2.0), moderate pollution (2.0 < PN ≤ 3.0) and heavy pollution (PN > 3.0) [19,34]. In this method, the synthetic pollution index focuses on the overall pollution situation caused by all the metals, considering not only the average single-factor pollution index but also its maximum values [35].

### 3.5. Assement of Potential Ecological Risk

The potential ecological risk index (RI) proposed by Hakanson is widely used in assessing heavy metals pollution [33], and the equations to calculate this index are as follows:

$$C_{f}^{i}=C_{s}^{i}/C_{n}^{i}$$

$$E_{r}^{i}=T_{r}^{i}\times P_{i}$$

$$\mathrm{RI}=\sum^{\text{​}}E_{r}^{i}$$

where $C_{f}^{i}$ is the single metal pollution factor; $C_{s}^{i}$ is the concentration of heavy metal in samples, mg/kg; $C_{n}^{i}$ is the background values of soil in Shanxi Province, mg/kg; $E_{r}^{i}$ is the monomial potential ecological risk coefficient; $T_{r}^{i}$ is the metal toxic response factor; according to Hakanson, the values for each element are as follows: Zn (1) < Cr (2) < Cu (5) = Ni (5) = Pb (5) < As (10) < Cd (30); RI is the potential ecological risk index. The categories of metal pollution according to the monomial potential ecological risk coefficient ($E_{r}^{i}$) are as follow: low risk ($E_{r}^{i}$ < 40), moderate risk (40 ≤ $E_{r}^{i}$ < 80), high risk (80 ≤ $E_{r}^{i}$ < 160), very high risk (160 ≤ $E_{r}^{i}$ < 320), extremely high risk ($E_{r}^{i}$ ≥ 320); The categories of metal pollution according to the potential ecological risk index (RI) are as follow: low risk (RI < 150), moderate risk (150 ≤ RI < 300), high risk (300 ≤ RI < 600), very high risk (RI ≥ 600) [17,18,19].

Comparing with other method of assessing heavy metals pollution to environment, Hakanson suggested that the potential ecological risk index is mainly related to the concentration, type, quantity, toxicity, and sensitivity of metal pollutants, etc. [36].

## 4. Results and Discussion

### 4.1. The Concentration of Heavy Metals in Sewage Sludge

The concentration of heavy metals was determined and is presented in Table 2. According to the mean values of concentration, the heavy metals were ranked in decreasing order as follows: Zn > Cr > Cu > Ni > Pb > As > Cd. The mean value of each heavy metal was 331.50 mg/kg, 147.72 mg/kg, 100.53 mg/kg, 34.28 mg/kg, 11.59 mg/kg, 11.56 mg/kg, and 0.65 mg/kg, respectively. The content of Zn was the highest, while that of Cd was the lowest. Because sewage sludge was collected from different regions, the concentrations of the heavy metals varied greatly. The concentrations of Zn, Cr, Cu, Ni, Pb, As and Cd, varied individually from 5.98 to 561.00 mg/kg, 1.49 to 312 kg/kg, 9.88 to 253 mg/kg, 0.75 to 84.88 mg/kg, 2.63 to 32.92 mg/kg, 6.61 to 23.90 mg/kg, and 0.27 to 0.87 mg/kg, respectively. Because sewage sludge samples were collected from different sites, the standard deviations of heavy metals contents changed greatly. In addition, regarding the differences between heavy metals contents in sewage sludge and the background values in shale, this variation may be attributed to the different anthropogenic sources in the study area [28,34].

Generally, the municipal wastewater consisted of industrial wastewater, domestic wastewater, and stormwater drains [37].

Metallurgy and coking give rise to local contamination of Cu, Zn, Ni, Cd and Cr [37,38,39]. Cd can also arise from the electronics industry [35]. The station of 2, 3, 4, 8 and 9 were close to metallurgy and coking industries, and the concentrations of Cu, Zn, Ni, Cd and Cr in these stations were higher than others. The level of Cd was also high for station 1, located near an electronics industrial park, which may have caused that. The contamination of Cr also could be caused by cement production, chemical engineering, as well as other industries such as painting, coating and leather tanning industries [40,41]. The station 3 had a high level of Cr, possibly caused by cement production, and station 1 and 8 had a high level of Cr, which may have been induced by the chemical engineering plant. Coal mining could cause As pollution at a high level [26]. As station 7 was the coal production base in China, the content of As in station 7 was higher than that in the others stations.

Traffic is a main pollution source for Pb [37]. The emission of Pb suspensions from gasoline enters into wastewater through runoff water [42,43]. Station 6, 3 and 5, which were traffic hubs in the studying area were all had high level of Pb.

Households can also contribute to heavy metals pollution. The usage of detergents containing arsenic is another reason for As pollution [37]. Stations 1, 2, 3 and 4, which are all located in the capital of the studying area, had high levels of As, possibly because of the domestic wastewater.

The heavy metals contents in the study stations were lower than those in the other regions in China, and only As was equal to the mean value of China. Additionally, compared with the threshold values of the Chinese Control Standards for Pollutants in Sludge from Agricultural Use (GB4284-84), the maximum concentrations of the heavy metals were all within the content limits permitted by the discharge standard. This fact illustrated that sewage sludge from the study stations can be directly applied for land use without reducing the content of heavy metals.

### 4.2. Geoaccumulation Index Values for Heavy Metals in Sewage Sludge

The values of I_geo_ for the seven heavy metals were listed in Table 3, and, on the basis of the classification by Muller, heavy metals in sewage sludge were divided into 5 pollution groups, class 0 to 4. According to the mean values of I_geo_, the samples of sewage sludge were enriched with heavy metals in the following order: Cd > Zn > Cu > As > Cr > Ni > Pb, and the pollution order of stations was 9 > 8 > 3 > 2 > 1 > 4 > 6 > 5 > 7.

The I_geo_ values of Pb at station 1, 2, 3, 4, 5, 7 and 8, Cu at station 5 and 7, Zn at station 7, Ni at station 1, 2, 3, 4, 5, 6 and 7, Cr at station 5, 6 and 7 and As at station 5 were less than zero, suggesting that these sites were not polluted by these metals. The I_geo_ values of Pb at station 6 and 9, Cu at station 3, 4 and 6, Zn at station 5, Ni at station 8 and 9, Cr at station 4 and As at station 1, 2, 3, 4, 6, 8 and 9, were between 0 and 1, indicating that the pollution level of these metals at these stations ranged from unpolluted to moderately polluted; The I_geo_ values of Cu at station 1 and 2, Zn at station 6, Ni at station 2, Cr at station 1, 2, 3, 8 and 9, As at station 7 and Cd at station 6 were between 1 and 2, depicting that the pollution level of these metals at these stations were moderate; The I_geo_ values of Cu at station 8 and 9 and Cd at station 5 and 7 were between 2 and 3 suggesting that Cu and Cd in these stations were polluted in moderately to heavily level; The I_geo_ values of Zn at station 3 and Cd at station 1, 2, 3, 4, 8 and 9 were more than 3, suggesting that the pollution level of these metals at these stations were heavily.

As other regions in China, heavy metals pollution in sewage sludge was not significant except for Cd, which might be caused by the rich coal resources in Shanxi, and the large number of coal industrial facilities, as the pillar industry in this region [11,44,45]. However, the heavy metal pollution in station 3, 8 and 9 are higher than other stations, which might be caused by the metallurgy industry in the areas of these stations [46].

### 4.3. Assessment of Heavy Metal Pollution

The PI of heavy metals was summarized in Table 4. The mean values of PI for the seven heavy metals were in the following decreasing order: Zn > Cu > Ni > Cr > As > Cd > Pb. According to the values of PI, sewage sludge in studying stations were unpolluted with most heavy metals, except for Cu at station 9, and Zn for station 3 and 8 were in low levels of contamination but higher than the limit of Chinese Control Standards for Pollutants in Sludge from Agricultural Use (GB4284-84). According to the values of PN, the stations were in the following decreasing order: 3 > 9 > 8 > 4 > 6 > 1 > 2 = 5 > 7, and the values of PN for station 1, 2, 4, 5, 6 and 7 were lower than 0.7, illustrating that sewage sludge at these stations was safe in terms of heavy metals release to the environment, and could be used directly for land. The values of PN for station 3, 8 and 9 were between 0.7 and 1, indicating that sewage sludge at these stations had dangerous levels of heavy metals, and, when applying the sewage sludge for land use, require attention to the heavy metals pollution issues.

Because of the complexity of sewage sludge, PI can’t reflect the pollution level better than PN, which combines the pollution levels of all metals, to accurately reveal the pollution levels of sewage sludge in a station [40]. Using PN, station 3, 8 and 9 were the most seriously polluted, possibly may because the areas where these stations located are more dominated by the chemical industry [47].

### 4.4. The Potential Ecological Risk

According to the Hakanson approach, the results of $E_{r}^{i}$ and RI for each studied station were show in Table 5.

By the mean values of $E_{r}^{i}$, heavy metals were sorted in the decreasing order: Cd > Cu > As > Ni > Cr > Zn > Pb. The values of $E_{r}^{i}$ for Pb, Cu, Zn, Ni, Cr and As in all stations were lower than 40, indicating that these stations didn’t have a potential ecological risk for these metals. The values of $E_{r}^{i}$ for Cd suggested that all stations had very high potential ecological risk for Cd, except for station 6 with moderate risk, and 5 and 7 with high risk. According to the RI values, stations can be sorted in the decreasing order: station 9 > 8 > 1 > 3 > 2 > 4 > 5 > 7 > 6, and they can be classified into 4 grades. For station 6, the value of RI was less than 150, indicating that it had low potential ecological risk; For station 2, 3, 4, 5, 7 and 8, the values of RI were between 150 and 300, indicating that these stations had moderate potential ecological risk; For station 1 and 9, the values of RI were between 300 and 600, indicating that these stations had high potential ecological risk.

For assessing heavy metals pollution to the environment, $E_{r}^{i}$ and RI are mainly related to the toxicity of heavy metals; the other indices do not focus on this aspect [40]. Because heavy metals have different toxicity, the ecological risk changes depending on the influence of the particular metals [1,41]. For this study, Cd was the critical influencing factor, and it contributed to the potential ecological risk for all of the stations.

## 5. Conclusions

The disposal situation of sewage sludge was investigated in Shanxi Province. To evaluate the risk of heavy metals in sewage sludge for land use, samples were collected from 9 WWPTs that produced more than 5000 tons per day of sewage sludge. The results indicated that land use, which accounted for 42.66% of sewage sludge, was the chief method of handling the increasing sewage sludge. The mean concentration of heavy metals decreased in the following order: Zn > Cr > Cu > Ni > Pb > As > Cd. The contents of Pb, Cu, Zn, Ni, Cr, As and Cd in sewage sludge were within standard limits of the Chinese Control Standards for Pollutants in Sludge from Agricultural Use (GB4284-84) and lower than those in other regions in China, except for As, which was equal.

According to the mean values of I_geo_, the heavy metals were ranked in the following order: Cd > Zn > Cu > As > Cr > Ni > Pb, with Cd pollution being the highest, Zn pollution being moderate and Pb, Cu, Ni, Cr and As pollution being lower. The values manifested that the pollution levels of Zn in station 3 and Cd in station 1, 2, 3, 4, 8 and 9 were heavily polluted; Cu in station 8 and 9, Zn in station 1, 2, 4, 8 and 9 and Cd in station 5 and 7 were moderately to heavily polluted, and other heavy metals accumulation were not significant. Heavy metal pollution in station 3, 8 and 9 are higher than other stations, and it was not as significant as that in other regions in China. By the mean values of PI, heavy metals were in the following decreasing order: Zn > Cu > Ni > Cr > As > Cd > Pb and all stations were not contaminated, but station 3 with Zn and station 8 with Zn and Cu were in a low level of contamination. According to the values of PN, the sewage sludge in all stations except for 3, 8 and 9 were safe for land use in terms of heavy metals pollution. Based on the mean values of $E_{r}^{i}$, which summed the toxicity of heavy metals, the heavy metals were decreasing in the following order: Cd > Cu > As > Ni > Cr > Zn > Pb and all metals except Cd did not have potential ecological risk. Stations were sorted according to the values of RI in decreasing order: 9 > 8 > 1 > 3 > 2 > 4 > 5 > 7 > 6 and the risk of station 1, 8 and 9 were considerably higher than the other stations. On the basis of $E_{r}^{i}$ and RI, Cd was the critical influencing factor that contributed to the potential ecological risk of all stations. All indices revealed that station 3, 8 and 9 had a higher risk of heavy metals pollution in sewage sludge for land use. In addition, Cd was the critical influencing factor contributing to the risk regarding the accumulation and toxicity of heavy metals. This might be caused by the metallurgy industry located near station 3, 8 and 9.

Land use of sewage sludge is the main disposal method in Shanxi and which utilizes the nutrients in sludge but also leads to an increased risk of heavy metals getting into soils. With the increasing amount of sewage sludge, it has become an important environmental problem [48,49].

To reduce the potential risk of heavy metals pollution in soil, a risk assessment is necessary. To mitigate this potential risk, appropriate treatment technologies such as electro-kinetic technology, bioleaching, chemical extraction and phyto-extraction should be used before sewage sludge is applied on land [50,51,52,53]. After application of one of these treatment methods, if the heavy metals content in sewage sludge remains at a high level, then it should be disposed by landfill or burning.

Furthermore, proper policies on land use of sewage sludge should be formulated based on the Chinese Control Standards for Pollutants in Sludge from Agricultural Use (GB4284-84). Although it has played an important role but it contains some defects for the current point of view. It was the strictest standard compared with similar standards worldwide, and this level of strictness is not essential [35,36]. To make better use of sewage sludge, the standard should be revised and the limit of heavy metals contents in sewage sludge should be lifted. And then, soil environmental capacity which is not included in the standard, should be added; because long-term use of sewage sludge on land will damage soil properties and increase the ecological risk to the environment. If the land already has high contents of heavy metals, then the soil environmental capacity should be assessed to decide whether to use sewage sludge on land continually.

## Figures and Tables

**Table 1:** Disposal situation of sewage sludge in Shanxi in 2011 ^1^.

Region	Land Use		Land Fill		Construction Matter Producing		Burning		Dumping	
	Total	Percentage	Total	Percentage	Total	Percentage	Total	Percentage	Total	Percentage
North of Shanxi	6305	9.54	56,772.95	85.90	1100.6	1.67	1911.3	2.89	0	0
Middle of Shanxi	127,950.2	72.90	40,287.2	22.95	21	0.01	7247.2	4.13	13.5	0
South of Shanxi	17,272	24.06	33,626.1	46.84	11,609	16.17	9287	12.94	0	0
Southeast of Shanxi	8383	13.64	52,290	85.09	19.1	0.03	756	1.23	8	0.01
Shanxi	159,910.2	42.66	182,976.25	48.81	12,749.7	3.40	19,201.5	5.12	21.5	0

^1^ The statistical statement of sewage sludge disposal in wastewater treatment plants in Shanxi in 2011.

**Table 2:** Concentrations of heavy metals (mg/kg) in sewage sludge from 9 WWTPs.

Station	Pb	Cu	Zn	Ni	Cr	As	Cd
1	4.13	109.88	379.00	18.50	169.88	11.88	0.87
2	2.63	115.13	381.75	84.88	169.00	10.01	0.66
3	12.75	77.38	561.00	32.50	312.00	9.82	0.69
4	3.25	84.25	436.38	30.00	87.50	11.14	0.69
5	6.50	9.88	98.50	8.25	29.50	6.61	0.52
6	32.92	66.09	203.42	29.04	76.23	8.34	0.27
7	2.99	11.95	5.98	0.75	1.49	23.90	0.45
8	14.25	177.25	531.13	66.00	239.25	8.85	0.82
9	24.88	253.00	386.38	38.63	244.63	13.48	0.83
Mean	11.59	100.53	331.50	34.28	147.72	11.56	0.65
Max	32.92	253.00	561.00	84.88	312.00	23.90	0.87
Min	2.63	9.88	5.98	0.75	1.49	6.61	0.27
SD	10.87	77.04	189.73	26.66	105.95	5.05	0.20
Mean values of China [35]	131	486	1450	77.5	185	11.5	2.97
GB4284-84 ^1^							
Ph < 6.5	1000	1500	3000	100	1000	75	20
Ph ≥ 6.5	300	800	2000	200	600	75	5

^1^ Chinese Control Standards for Pollutants in Sludge from Agricultural Use.

**Table 3:** Geoaccumulation index values for heavy metals in sewage sludge.

Station	I_geo_							
	Pb	Cu	Zn	Ni	Cr	As	Cd	Mean
1	−2.00	1.39	2.49	−1.02	1.12	0.66	3.46	0.87
2	−2.65	1.45	2.50	1.18	1.12	0.42	3.06	1.01
3	−0.37	0.88	3.06	−0.21	2.00	0.39	3.12	1.27
4	−2.34	1.00	2.69	−0.32	0.17	0.57	3.12	0.70
5	−1.34	−2.09	0.55	−2.18	−1.40	−0.18	2.71	−0.56
6	1.00	0.65	1.59	−0.37	−0.03	0.15	1.76	0.68
7	−2.47	−1.81	−3.50	−5.65	−5.71	1.67	2.49	−2.14
8	−0.21	2.08	2.98	0.82	1.62	0.24	3.36	1.55
9	0.59	2.59	2.52	0.04	1.65	0.85	3.39	1.66
Mean	−1.09	0.68	1.65	−0.86	0.06	0.53	2.94	-
Max	1.00	2.59	3.06	1.18	2.00	1.67	3.46	-
Min	−2.65	−2.09	−3.50	−5.65	−5.71	−0.18	1.76	-
SD	1.38	1.61	2.08	2.05	2.41	0.52	0.55	-

“-” means not calculated.

**Table 4:** Results of single factor pollution index (PI) and the Nemerow’s synthetic pollution index (PN) for heavy metals in sewage sludge for land use.

Station	PI							PN
	Pb	Cu	Zn	Ni	Cr	As	Cd	
1	0.01	0.44	0.76	0.19	0.28	0.16	0.17	0.12
2	0.01	0.46	0.76	0.85	0.28	0.13	0.13	0.09
3	0.04	0.31	1.12	0.33	0.52	0.13	0.14	0.79
4	0.01	0.34	0.87	0.30	0.15	0.15	0.14	0.62
5	0.02	0.04	0.20	0.08	0.05	0.09	0.10	0.09
6	0.11	0.26	0.41	0.29	0.13	0.11	0.05	0.30
7	0.01	0.05	0.01	0.01	0.00	0.32	0.09	0.06
8	0.05	0.71	1.06	0.66	0.40	0.12	0.16	0.75
9	0.08	1.01	0.77	0.39	0.41	0.18	0.17	0.77
Mean	0.04	0.40	0.66	0.34	0.25	0.15	0.13	0.40
SD	0.04	0.31	0.38	0.27	0.18	0.07	0.04	0.33

**Table 5:** Potential ecological risk assessment results of heavy metals in sewage sludge for land use.

Station	$E_{r}^{i}$							RI
	Pb	Cu	Zn	Ni	Cr	As	Cd	
1	1.40	23.99	5.97	3.09	6.14	13.05	256.62	310.27
2	0.89	25.14	6.01	14.19	6.11	11.00	194.41	257.76
3	4.34	16.89	8.83	5.43	11.28	10.79	203.82	261.40
4	1.11	18.40	6.87	5.02	3.16	12.24	203.90	250.69
5	2.21	2.16	1.55	1.38	1.07	7.26	153.31	168.94
6	11.20	14.43	3.20	4.86	2.76	9.16	79.41	125.02
7	1.02	2.61	0.09	0.12	0.05	26.26	131.76	161.93
8	4.85	38.70	8.36	11.04	8.65	9.73	239.71	321.03
9	8.46	55.24	6.08	6.46	8.85	14.81	244.71	344.61
Mean	3.94	21.95	5.22	5.73	5.34	12.70	189.74	244.63
SD	3.70	16.82	2.99	4.46	3.83	5.55	58.39	77.06
